# Microglial P2Y12 receptor regulates ventral hippocampal CA1 neuronal excitability and innate fear in mice

**DOI:** 10.1186/s13041-019-0492-x

**Published:** 2019-08-19

**Authors:** Jiyun Peng, Yong Liu, Anthony D. Umpierre, Manling Xie, Dai-Shi Tian, Jason R. Richardson, Long-Jun Wu

**Affiliations:** 1 Institute of Life Science, Nanchang University, Nanchang, 330031 China; 2 Department of Neurology, Mayo Clinic, 200 First Street SW, Rochester, MN 55905 USA; 3 Department of Neurology, Tongji Medical College, Huazhong University of Science and Technology, Wuhan, 430030 China; 4 Departments of Environmental Health Sciences, Florida International University, Miami, FL 33199 USA; 5 Department of Neuroscience, Mayo Clinic, Jacksonville, FL 32224 USA; 6 Department of Immunology, Mayo Clinic, Rochester, MN 55905 USA

**Keywords:** Microglia, P2Y12 receptors, Hippocampus, Conditional knockout, Innate fear, Anxiety

## Abstract

The P2Y12 receptor (P2Y12R) is a purinoceptor that is selectively expressed in microglia in the central nervous system. As a signature receptor, microglial P2Y12R mediates process chemotaxis towards ADP/ATP gradients and is engaged in several neurological diseases including chronic pain, stroke and seizures. However, the role of microglial P2Y12R in regulating neuronal excitability and innate behaviors is not fully understood. Here, we generated P2Y12-floxed mice to delete microglial P2Y12R beginning in development (CX_3_CR1^Cre/+^:P2Y12^f/f^; “constitutive knockout”), or after normal development in adult mice (CX_3_CR1^CreER/+^:P2Y12^f/f^; “induced knockout”). Using a battery of behavioral tests, we found that both constitutive and induced P2Y12R knockout mice exhibited innate fear but not learned fear behaviors. After mice were exposed to the elevated plus maze, the c-fos expression in ventral hippocampus CA1 neurons was robustly increased in P2Y12R knockout mice compared with wild-type mice. Consistently, using whole cell patch clamp recording, we found the excitability of ventral hippocampus CA1 neurons was increased in the P2Y12R knockout mice. The results suggest that microglial P2Y12R regulates neuronal excitability and innate fear behaviors in developing and adult mice.

## Introduction

Microglia are the resident immune cells in the central nervous system (CNS) and play key roles in health and disease [1–5]. Microglia have been reported to be important for brain development, particularly circuit formation, via pruning excessive synapses as well as inducing spine formation [6–8]. In addition, multiple lines of evidence have shown that microglial dysfunction leads to neurological disorders. For example, TREM2 and DAP12 are selectively expressed in CNS microglia and their mutations were linked to Nasu-Hakola disease (NHD), a condition that results in dementia [9–11]. In line with this, genome-wide association studies have identified that TREM2 rare variants significantly increase the risk of developing Alzheimer’s diseases [12, 13]. Microglial CSF1 receptors are critical for microglia survival [14] and their mutations in the tyrosine kinase domain causes Hereditary Diffuse Leukoencephalopathy with Spheroids (HDLS), a rare autosomal dominant disease with similar neural degenerative pathology as NHD [15]. Interestingly, mice deficient in Hoxb8, a homeobox gene expressed in myeloid derived microglia, showed compulsive grooming, a phenotype mimicked the obsessive-compulsive disorder (OCD) [16]. Similarly, mice with microglial deficiency of progranulin (GRN), a protein important for frontotemporal dementia, also displayed increased self-grooming [17, 18]. Considering the seemingly contradictory results on microglial function in learning and memory [19, 20], however, the role of microglia in adult brain homeostasis and behavioral adaptations is still largely unexplored.

The unique feature of microglia is that they have dynamic processes that survey the brain parenchyma constantly [21, 22]. Microglia make rapid chemotactic responses by extending processes to the injury site or hyperactive neurons, which serve as sources of ADP/ATP and activate microglial P2Y12 receptors (P2Y12R) [23, 24]. P2Y12R is highly and exclusively expressed in microglia in healthy brain, making it a so-called “signature receptor” in microglia [25–28]. Although P2Y12R seems to not be involved in the basal motility of microglial processes, it is well established to control acute microglial process chemotaxis towards an ATP source [29]. In addition, P2Y12R is critical for microglial cell body translocation in response to seizures or sensory deprivation, which leads to microglial landscape changes in vivo [30, 31]. Therefore, microglial P2Y12R regulation of process dynamics and soma translocation might be important for neuronal activity and behavioral adaptations. Indeed, a recent study found that P2Y12R is necessary for microglial responses to monocular deprivation and ocular dominance plasticity in the visual cortex [32]. However, it is still unknown how microglial P2Y12R may regulate neuronal network function and possible innate behaviors in adult mice.

In this study, we generated P2Y12-floxed mice (P2Y12^f/f^) and then crossed these mice with CX_3_CR1^Cre^ or CX_3_CR1^CreER^ lines to obtain the constitutive or induced knockout (KO) of P2Y12R in microglia respectively. We found that both the constitutive and induced P2Y12R KO mice exhibited anxiety-like behaviors. In addition, neuronal excitability was increased in ventral hippocampal CA1 neurons in P2Y12R KO mice. These results suggest that microglial P2Y12R is required to maintain neuronal network homeostasis and thus regulates innate fear behaviors.

## Results

### Conditional deletion of P2Y12 receptors from microglia

**Fig. 1:**
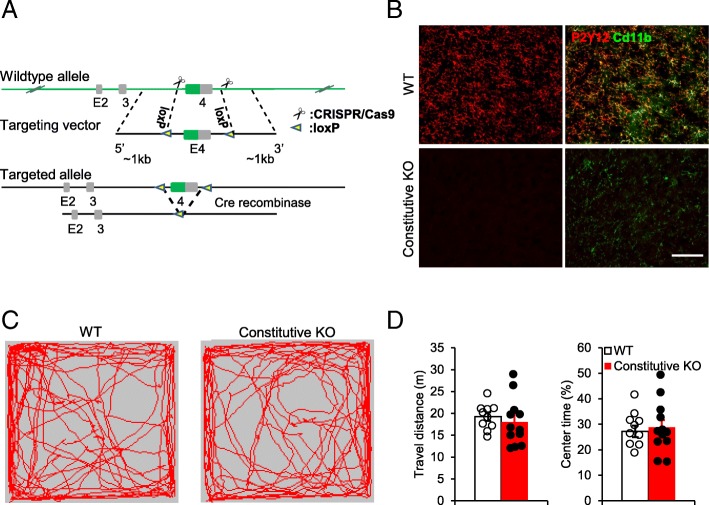
Constitutive knockout of microglial P2Y12R using P2Y12-floxed mice. **a** Schematic of the strategy used to generate the loxP flanked *p2ry12* mice. **b** Representative immunostaining images showing P2Y12R was expressed in Cd11b + cells in WT, but absent in P2Y12^f/f^:CX_3_CR1^Cre/+^ (constitutive KO) mice. Scale bar: 50 μm. **c** Open field test locomotor activity between constitutive microglial P2Y12R KO (*n* = 12) and WT control (*n* = 10) groups. **d** The pooled results from open field test indicated similar locomotor activity (left) and center area exploration (right) between WT and constitutive KO groups

The Cre-loxP strategy was used to conditionally delete the *p2ry12* gene from microglia. To this end, the P2Y12-floxed mice were generated by the CRISPR/Cas9 technique. Exon 4 of the *p2ry12* gene was flanked with one of the loxP fragments inserted into intron 3 and the other inserted downstream of 3’UTR of *p2ry12* (Fig. 1a). The P2Y12-floxed mice were then crossed with the CX_3_CR1^Cre/+^ mice to obtain the P2Y12^f/f^:CX_3_CR1^Cre/+^ (constitutive KO) mice. Immunostaining results showed that P2Y12R expression was completely removed from the constitutive KO mice in the adult brain (Fig. 1b).

To evaluate the possible behavioral alterations when P2Y12R is deficient, we tested the spontaneous activities of the constitutive KO mice in the open field. We found that the constitutive P2Y12R KO mice showed normal total travel distances (18.00 ± 2.06 m, *n* = 12) compared to WT control (19.26 ± 0.98 m, *n* = 10) (Fig. 1c-d). In addition, the constitutive KO mice showed similar center area exploration compared with WT controls (Fig. 1c-d). Therefore, constitutive deficiency of microglial P2Y12R does not affect normal locomotor activities in mice.

### Enhanced innate but not learned fear responses in microglial P2Y12 receptor knockout mice

**Fig. 2:**
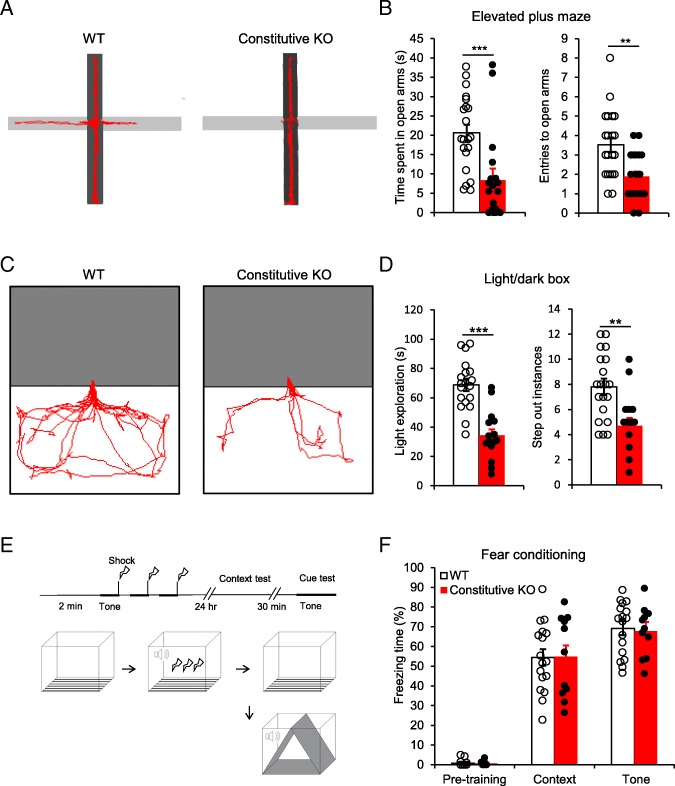
Microglial P2Y12R deficiency enhances innate, but not learned fear responses. **a** Elevated Plus Maze (EPM) evaluation of anxiety between constitutive microglial P2Y12R KO (*n* = 19) mice and WT controls (*n* = 21). **b** The constitutive KO mice spent less time in the open arms and were less likely to enter into the open arms than control mice. **c** Light/dark box testing evaluation of anxiety between constitutive KO mice (*n* = 15) and WT controls (n = 19). **d** Constitutive KO mice spent less time exploring the lighted side and stepped out less from the dark box. **e** Protocol for fear conditioning training and tests. Mice were foot shocked at the last 2 s of a 30 s auditory tone, the tone and foot shock pair were repeated for 3 times. Freezing responses to the context and tone stimulus were measured in the next day. **f** The microglial P2Y12R KO mice showed similar freezing responses to the training context and paired auditory tone stimulus as the WT control mice (*n* = 17 for the WT control, *n* = 11 for the constitutive KO group). **p < 0.01, ***p < 0.001, t-test and U-test. Data are presented as the mean ± SEM

Next, we evaluated mouse innate fear behaviors in the elevated plus maze (EPM) test. We found that the constitutive KO mice spent significantly less time (constitutive KO, 8.41 ± 2.57 s, *n* = 19) exploring the open arms compared to WT controls (WT, 20.66 ± 2.09 s, *n* = 21, *p* < 0.001). In addition, the constitutive KO mice made fewer entries into the open arms (constitutive KO, 1.89 ± 0.33; WT, 3.86 ± 0.37; *p* < 0.01) (Fig. 2a-b). These results suggest that microglial P2Y12R deficiency increases innate fear in mice.

To further investigate innate fear in mice, we conducted behavior tests using the light/dark shuttle box. During 5 min testing, WT mice spent 68.79 ± 3.87 s to re-explore the light box and stepped out of the dark box 7.8 ± 0.60 times (*n* = 19). Compared with WT mice, the constitutive KO mice spent only 34.47 ± 3.95 s on the lighted side (*p* < 0.001). In addition, the constitutive KO mice were less likely to step out of the dark box (4.71 ± 0.64 times, *n* = 15, *p* < 0.01) (Fig. 2c-d). Taken together, these results indicate that the mice with constitutive P2Y12R deficiency in microglia have enhanced innate fear responses.

To investigate whether microglial P2Y12R is required for fear learning, we performed fear conditioning using a foot-shock paradigm. Both contextual fear memory and auditory tone associated fear memory were tested 24 h after the conditioning training. When the mice were returned to the training box where they received electric foot shock (Unconditioned Stimulus, US), the mice from WT control (*n* = 17) and constitutive KO (*n* = 11) groups displayed similar freezing time during the 3 min test period. In a new environment, the auditory tone (Conditioned Stimulus, CS) that was previously paired with the foot shock induced similar freezing responses in the two groups as well (Fig. 2e-f). The results suggest that microglial P2Y12R deficiency did not affect conditional or learned fear responses.

### Increased c-fos expression in ventral hippocampal CA1 in microglial P2Y12 receptor knockout mice

**Fig. 3:**
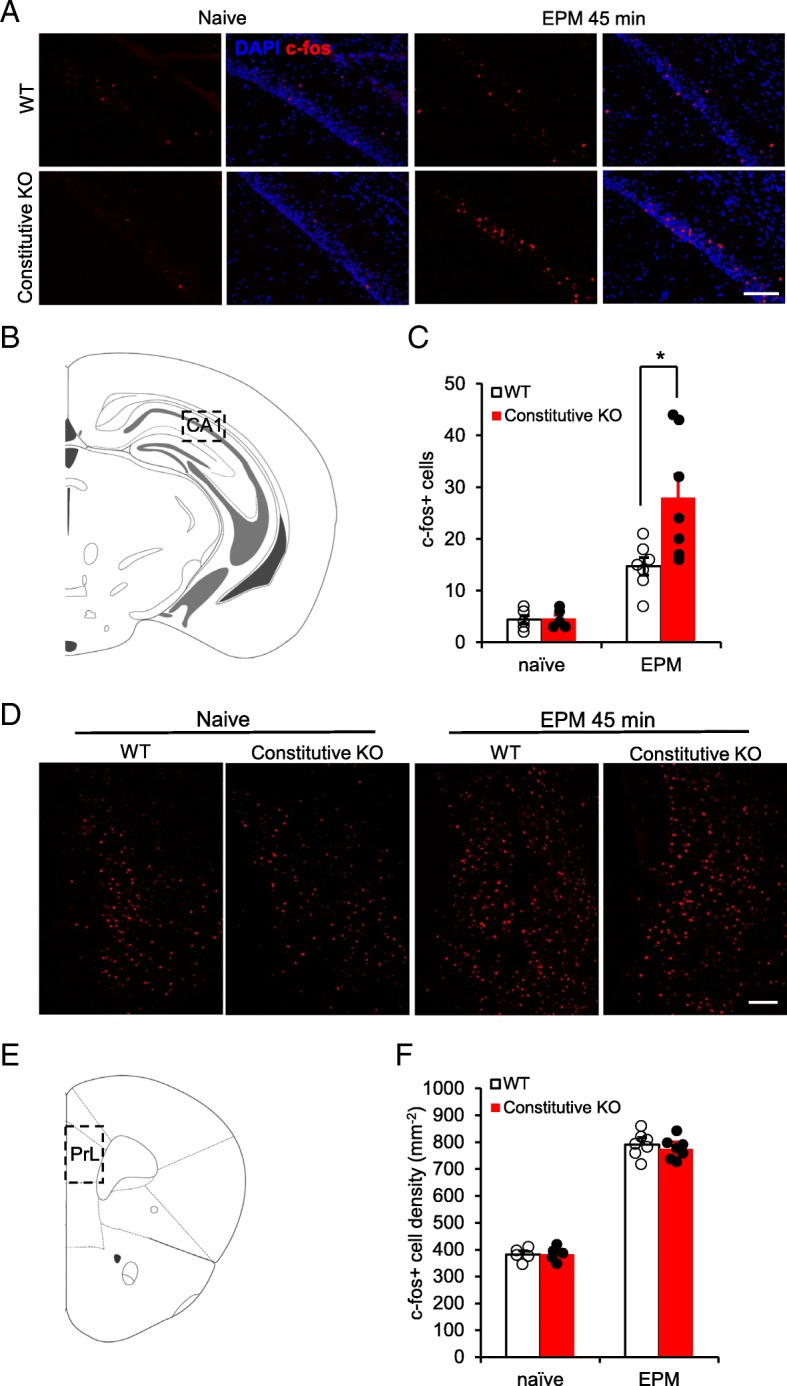
Microglial P2Y12R deficiency enhances the c-fos expression in the ventral hippocampus in response to elevated plus maze. **a** C-fos staining showing more activated neurons from vHPC CA1 in microglial P2Y12R deficiency group after exposing to EPM. **b** Diagram showing the CA1 area investigated in the ventral hippocampus. **c** Quantification of c-fos^+^ cells in the CA1 region indicates equivalent c-fos expression levels in WT and constitutive KO animals prior to EPM exposure (naive state). Enhanced c-fos activation occurs in the constitutive KO group after performing in the EPM, when compared to WT controls. **d** c-fos staining showing increased c-fos expression in the prefrontal cortex in both WT and constitutive KO mice after exposure to the EPM environment. **e** Diagram showing the prelimbic area studied in mPFC. **f** Quantification of c-fos^+^ cells in the prelimbic area indicates equivalent c-fos expression levels in WT and constitutive KO animals prior to EPM exposure (naive state). After EPM exposure, c-fos activation is enhanced in both control and constitutive KO groups and does not differ between groups. *p < 0.05, t-test. All data are presented as the mean ± SEM. Scale bar: 50 μm

Ventral hippocampus (vHPC) and medial pre-frontal cortex (mPFC) are known to be important for innate fear and anxiety behaviors [33]. To investigate how microglial P2Y12R deficiency alters the neuronal circuits for innate fear behaviors, we examined the c-fos expression in vHPC and mPFC at 45 min after the mice were exposed to EPM (10 min trial). At baseline (before exposure to the EPM), there was no difference in the number of c-fos positive cells in P2Y12R deficiency and WT mice in the vHPC (Fig. 3a-c, n = 5 images from 2 mice for each group). After EPM exposure, both P2Y12R constitutive KO and WT mice demonstrated c-fos positive cell increases in the vHPC compared to naive mice. However, there were significantly more c-fos positive cells in constitutive KO mice when compared to that in the WT mice (*n* = 7 images from 3 mice for each group, *p* < 0.05). In the mPFC, exposure to the EPM context also increased the number of c-fos positive cells, but there was no difference between WT and constitutive P2Y1R2 KO groups (Fig. 3d-f). These results suggest that abnormal neuronal excitability may develop in the vHPC of P2Y12R deficient mice following exposure to innate fear paradigms.

### Increased neuronal excitability in ventral hippocampal CA1 neurons in microglial P2Y12 receptor knockout mice

**Fig. 4:**
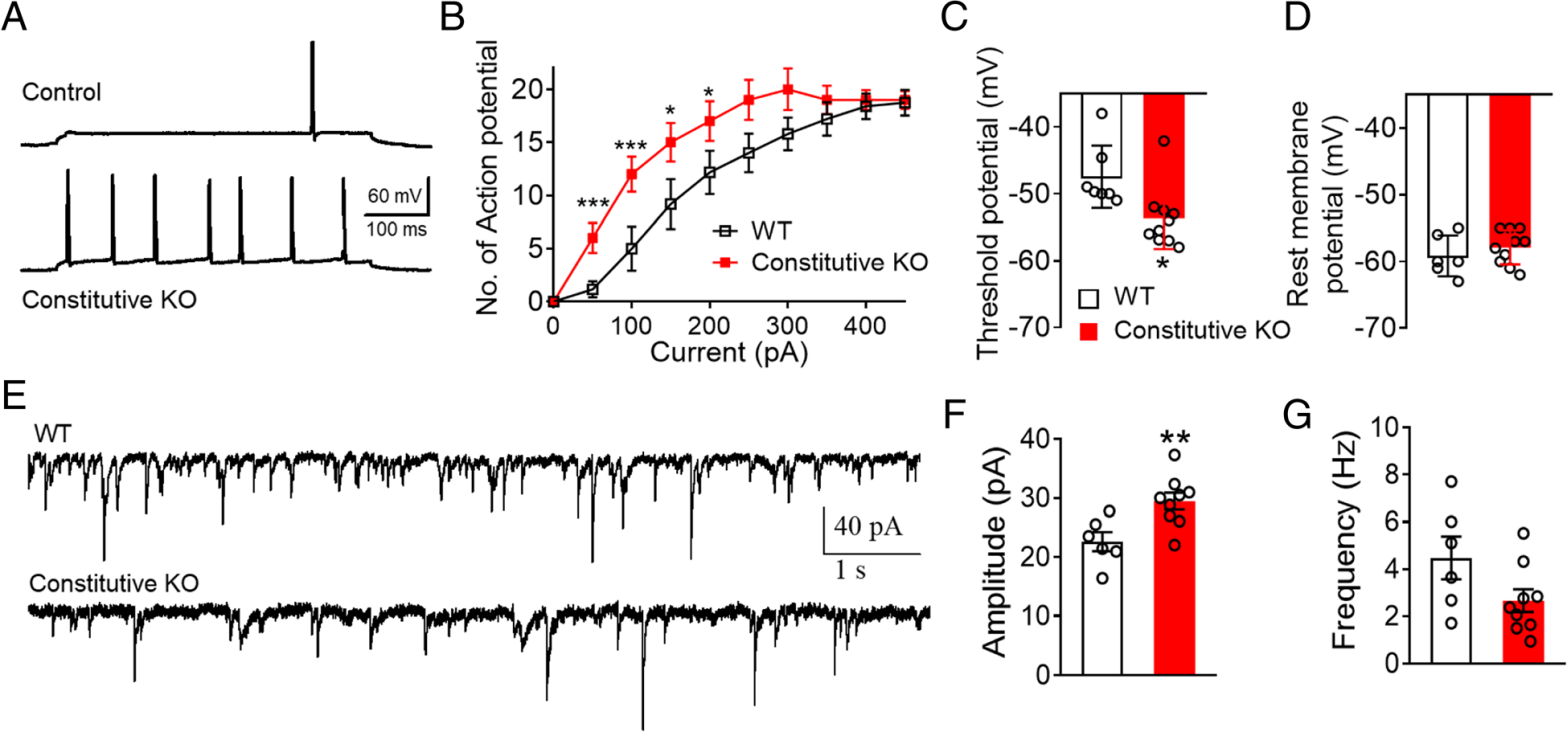
Microglial P2Y12R deficiency enhances the excitability of the hippocampal CA1 pyramid neurons. **a** Representative firing patterns of CA1 pyramidal neurons in response to 500 ms, 50 pA current steps in constitutive microglial P2Y12R KO and WT mice. **b** Input/output curve displaying the number of action potentials as the input current increases for each group. **c** The voltage threshold of action potential firing was significantly reduced in P2Y12R constitutive KO mice compared with WT mice. **d** There is no difference in resting membrane potential between the two groups. **e** Representative spontaneous excitatory postsynaptic currents (sEPSCs) from both groups. **f** and **g** sEPSC amplitude (**f**) and frequency (**g**) for the indicated groups *p < 0.05, **p < 0.01, ***p < 0.001 (t-test), *n* = 11 neurons from 3 mice in constitutive KO group and *n* = 8 neurons from 3 mice in WT group. All data are presented as the mean ± SEM

The increased c-fos expression in vHPC neurons could be due to enhanced innate excitability or stronger input projections to the vHPC. To differentiate between the two possibilities, we performed whole-cell patch clamp recordings from CA1 pyramidal neurons. Constitutive P2Y12R KO mice and their non-Cre littermatesWT mice were used in these studies. We found that the neurons from constitutive P2Y12R KO mice fired more action potentials (AP) compared to the WT group in response to current injection (Fig. 4a and b). Although their resting membrane potentials are comparable, neurons from the constitutive P2Y12R KO mice had a lower threshold for action potential firing compared with WT groups (*n* = 8 neurons from 3 mice for each group, *p* < 0.05, Fig. 4c and d). In addition, when spontaneous excitatory postsynaptic currents (sEPSCs) were recorded in those CA1 pyramidal neurons, we found the sEPSC amplitude in the constitutive KO group was significantly increased (Fig. 4e and f), while the event frequency was not altered (Fig. 4g). Taken together, these results indicate that abnormal CA1 pyramidal neuronal excitability in the vHPC may contribute to the heightened innate fear responses in microglial P2Y12R deficient mice.

### Increased innate fear responses and neuronal excitability in induced P2Y12 receptor knockout mice

**Fig. 5:**
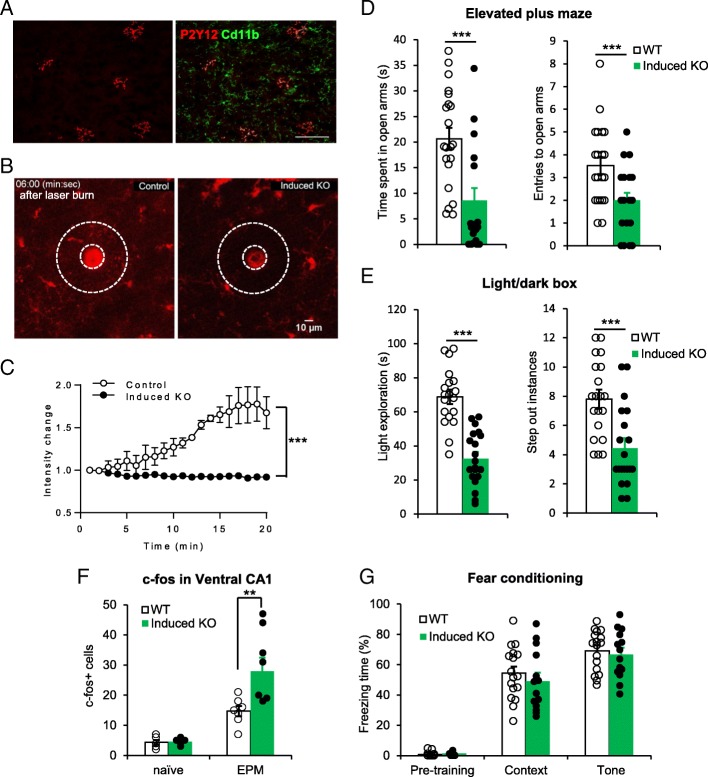
Induced knockout of microglial P2Y12 receptors enhances innate fear responses and c-fos expression. **a** Representative immunostaining images showing P2Y12R loss in most (85.9 ± 2.5%, *n* = 6 mice) of Cd11b + microglia cells after tamoxifen treatment in the adult P2Y12^f/f^:CX_3_CR1^CreER/+^ mice (Induced KO). **b** Representative images of microglial process chemotaxis 6 min after laser burn injury in vivo in induced microglial P2Y12R KO cortex (P2Y12^f/f^:ROSA^tdTomato/+^:CX_3_CR1^CreER/+^) and control (ROSA^tdTomato/+^:CX_3_CR1^CreER/+^) mice. **c** Intensity changes within the area surrounding the laser burn core (white ring area in panel B) after local injury. (*n* = 3 mice for each group. ***p < 0.001, two-way ANOVA.) **d** The induced P2Y12R KO mice (*n* = 18) showed decreased open arm time and entries in the EPM, compared with WT mice (*n* = 21). **e** The adult-induced KO mice (*n* = 20) showed less lighted side exploration time and stepped out less times from the dark enclosure in the light/dark box test, compared with WT mice (*n* = 19). The WT control in D-E were the same in Fig. 2 since the experiments were run together. **f** Quantification of c-fos^+^ cells in the ventral hippocampal CA1 region indicates equivalent c-fos expression levels in WT and induced KO animals prior to EPM exposure (naive state, *n* = 5 images from 2 mice for each group). Enhanced c-fos activation occurs in the induced KO group after performing in the EPM, when compared to WT controls (*n* = 7 images from 3 mice for each group). **g** 24 h after fear conditioning training, the adult-induced KO mice showed similar freezing responses to the training context and paired auditory tone stimulus as the WT control mice (*n* = 17 for the control, *n* = 13 for the induced KO group). **p < 0.01, ***p < 0.001, t-test or U-test. All data are presented as the mean ± SEM

Since microglia are known to participate in the neuronal synaptic maturation [7, 8], the behavioral alteration in constitutive P2Y12R KO mice could be due to the role of microglial P2Y12R in brain development. To rule out this possibility, we crossed the P2Y12-floxed mice with the CX_3_CR1^CreER/+^ mice to obtain the inducible KO mice, P2Y12^f/f^:CX_3_CR1^CreER/+^. Tamoxifen was given (TM, 8 × 100 mg/kg/48 h, i.p.) at 8 weeks after birth to inducibly delete P2Y12R from microglia in adult mice (induced P2Y12 KO). Immunostaining results showed P2Y12R was depleted in 85.9 ± 2.5% of Cd11b + microglial cells in the induced KO mice (Fig. 5a). To confirm the functional loss of microglial P2Y12 in the induced KO mice after TM treatment, we examined microglial process chemotaxis to the laser injury using two photon imaging. Microglia were labeled with tdTomato by crossing P2Y12^f/f^:CX_3_CR1^CreER/+^ mice or P2Y12^+/+^:CX_3_CR1^CreER/+^ mice with ROSA-tdTomato mice. We were able to visualize microglial process chemotaxis towards laser injury in ROSA^tdTomato/+^:P2Y12^+/+^:CX_3_CR1^CreER/+^ mice, while the process extension responses in ROSA^tdTomato/+^:P2Y12^f/f^:CX_3_CR1^CreER/+^ mice was largely impaired (Fig. 5b-c). These results demonstrated that in induced P2Y12 KO mice, microglia lost functional P2Y12R and thus cannot exhibit process chemotaxis toward laser injury as expected.

We then conducted the same EPM and light/dark box tests with the adult induced P2Y12R KO mice. In the EPM test, the induced KO mice spent 8.59 ± 2.42 s (*n* = 18, *p* < 0.001, compared with WT) to explore the open arms, and stepped into the open arms 1.97 ± 0.33 times (*p* < 0.001, compared with WT) (Fig. 5d). In the light/dark box test, the induced KO mice stepped out of the dark box 4.45 ± 0.68 times (*n* = 20, *p* < 0.001, compared with WT), the time spent in the light box was 32.55 ± 3.92 s (*p* < 0.001, compared with WT) (Fig. 5e). Therefore, the behavioral abnormalities of the adult induced P2Y12R KO mice were similar as those in constitutive KO mice. Parallel to the increased innate fear responses, EPM exposure similarly induced c-fos expression in neurons in the induced KO mouse to a greater degree than WT controls (Fig. 5f). We then examined fear learning behaviors in induced P2Y12R KO mice. We found that similar to constitutive P2Y12R KO mice, the learned fear responses in the induced KO mice were similar to WT mice (Fig. 5g). Therefore, inducible deletion of P2Y12R from microglia in adult mice increases neuronal immediate early gene expression and innate fear behaviors. The results suggest that microglial P2Y12R dependent purinergic signaling is constantly required for the homeostasis of neural circuit function underlying innate fear responses in adult mice.

## Discussion

In the current study, using microglial specific P2Y12R KO mice, we found that P2Y12R is necessary for maintaining neuronal circuit homeostasis in innate fear. Although microglial processes are constantly surveying the brain parenchyma, the role of microglia under physiological conditions in the adult brain is debated. With microglial ablation in the whole brain using csf1R inhibitor, Elmore et al. claimed that the mice did not have behavioral abnormality under open field, EPM and rotarod tests [20]. However, using the CX_3_CR1-CreER/iDTR ablation model, Parkhurst et al. found that motor learning dependent synapse formation requires microglia, and inter-session motor improvement on rotarod training was disrupted with microglia ablation [19]. Our previous study also confirmed that microglia-depleted mice showed normal motor performance but impaired learning effects on the second day of testing [34]. Therefore, it seems that microglia-depleted mice are viable without microglia, but impaired in their motor learning.

Microglia-neuronal communication may include several aspects such as physical contact between microglia processes and neuron elements, phagocytosis, release of BDNF and cytokines [2]. P2Y12R is critical for microglial process extension toward ATP gradients during brain injury [23] or to hyperactive neurons in seizures [24]. Microglial P2Y12R also controls microglial landscape changes in response to neuronal activity changes such as sensory deprivation or seizure induction [30]. The results suggest that microglial P2Y12R might be beneficial in dampening neuronal activity by increasing microglia-neuron interaction [24]. On the other hand, microglial P2Y12R are reported to participate in neuropathic pain [35–37] and ischemic stroke [38, 39]. At the cellular level, activation of microglial P2Y12R may lead to the release of proinflammatory cytokines and chemokines [40]. Therefore, it is understandable if the phenotype of P2Y12R KO mice differs from microglia-depleted mice.

Innate fear response in certain situations, such as the exposure to predators or height, is a genetic inherited protective reaction that benefits the animal’s survival during evolution [41]. However, strong fear emotion may cause anxiety and lead to post traumatic stress disorder (PTSD) [42, 43]. Therefore, proper neural wiring in innate fear circuits is critical for long-term survival and mental health [44]. The projection from vHPC to mPFC is involved in anxiety-like responses in mice [33, 45]. Padilla-Coreano et al. showed that optogenetic inhibition of vHPC to mPFC projections increased open arm activity in the EPM test [33]. Consistent with that notion, we found that enhanced vHPC neuronal activity (indicated by c-fos staining) and increased neuronal firing (shown by electrophysiological recordings) were correlated with increased innate fear behaviors (shown by decreased open arm activity) in P2Y12R KO mice. Therefore, our results suggest that microglial P2Y12R deficiency sensitized the circuit underlying the innate fear responses. The hippocampus has dynamic neural structure plasticity. Microglia deficient in P2Y12R may lose the ability to interact with neuronal elements and subsequently regulate plasticity. However, the molecular mechanisms underlying regulation of hippocampal circuits by microglial P2Y12R need further study. In sum, our present study provides evidence indicating that microglial P2Y12R participates in the maintenance of neuronal circuit homeostasis and innate fear behaviors.

## Methods

### Animals

The described procedures were approved by Institutional Animal Care and Use Committee (IACUC) at Nanchang University and Mayo Clinic. We followed the guidelines set forth by the Guide of the Care and Use of Laboratory Animals 8th Edition. P2Y12-floxed mice were designed and produced by Biocytogen Co., Ltd. (Beijing, China) and then bred at Mayo Clinic. CX_3_CR1-CreER mice were originally provided by Dr. Wen-Biao Gan in NYU. CX_3_CR1-Cre and ROSA-tdTomato mice were obtained from The Jackson Laboratory. All mice are on aC57BL/6 background. Only male mice were used for the whole study. Litter mates were used as KO or WT control. The experimenters were blinded to genotypes. Mice were group housed (4–5 per cage) in 12/12 light/dark cycle, 23 ± 1 °C vivarium environment. Food and water were available ad libitum.

### Behavioral measurement

The *open field* was custom made using light grey plastic boards with 40 (L) × 40 (W) × 20 (H) cm dimensions. Mouse cages were transferred to the test room for 30 min before the experiment started. Two mice from the same cage were tested simultaneously in two separated boxes. Mice were put in one of the corners with head to the corner and allowed to explore the box freely. The mouse activities were video monitored for 5 min. The mouse movement was offline tracked and analyzed using custom made software. The same software was used for the elevated plus maze and light/dark box analysis as well [46].

The *elevated plus maze* was custom made using light grey plastic boards. The arm length is 35 cm, lane width is 5 cm. The closed arm wall is 15 cm. The open arms have a small wall with 0.5 cm height to decrease falls. The maze is elevated 65 cm from the ground. The animals were transferred to the test room for 30 min prior to the experiment start to habituate to the environment. Mice were placed at the center of the plus maze gently with head to the open arm. Mice were allowed to explore for 5 min. Mice activities were video taped for off-line analysis.

The *light/dark box* contained two equal size chambers with 40 (L) × 20 (W) × 20 (H) cm dimensions. The two parts were separated with a wall of 20 cm height and connected with a 5 × 5 cm open gate. The light part was open on the top and the dark part was full covered with top lid. All the floors, walls and the top lid were made with the same light grey plastic boards. Mice were transferred to the test room for 30 min prior to the experiment start. Mice were put in one of the corners of the light box with head to the corner. Mice activities were video monitored. The recording ended at 5 min after the mice entered into the dark part for the first time.

The *fear conditioning tests* were conducted with the Video Freeze® fear conditioning system (Med Associates Inc., USA). Mice were transferred to the test room for 60 min habituation in the first day. Up to four mice were tested simultaneously in four test chambers. In the first training day, the chambers were cleaned with 70% alcohol. Mice were allowed to explore the chamber for 2 min, then a 30 s tone (85 dB, 700 Hz) was played. During the last 2 s, a mild foot shock (0.45 mA) was delivered. The tone-shock pairs were presented for 3 times with 15 s intervals. Mice were kept in the chamber for another 60 s after the last shock. Mice were tested for context fear memory after 24 h. Mice were placed back to the same chamber and allowed to explore for 3 min. The total freezing and motion time were recorded by the system. Mice were then transferred to another room for 30 min. The chamber context was changed with new floor and walls and wiped with bleach. Mice were then put back to a different chamber. After 2 min, the same tone was played for 3 min. The total freezing and motion time were recorded for each period [47].

### In vivo two-photon imaging of microglia

Mice were implanted with a 3-mm glass coverslip at about − 2.5 posterior and ± 2 mm lateral to bregma to replace the skull. Mice were maintained under anesthesia with Isoflurane (1.5% in O_2_) during imaging. In vivo imaging was performed by using a two-photon microscope (Scientifica) with a Ti:Sapphire laser (Mai Tai; Spectra Physics) tuned to 900 nm with a 40× water-immersion lens (0.8 NA; Olympus). Fluorescence was detected using two photomultiplier tubes in whole-field detection mode and a 565-nm dichroic mirror with 525/50-nm (green channel) and 620/60-nm (red channel) emission filters. The laser power was maintained at 30–40 mW, and images were collected from 60 μm to 100 μm into the brain. For imaging microglial dynamic from each mouse, z stack images were collected at 2-μm intervals in several FOVs. To perform a general laser injury, we focused the laser 66× and parked it at 250 mW at 900 nm for 1–3 s.

### Electrophysiology

Transverse acute hippocampal slices (350 μm) were cut in chilled (2–4 °C) cutting solution containing (in mM): 185 sucrose, 2.5 KCl, 1.2 NaH_2_PO_4_, 25 NaHCO_3_, 25 glucose, 10 MgCl_2_, 0.5 CaCl_2_. The slices were then transferred to an incubator with artificial cerebrospinal fluid (ACSF, in mM): 130 NaCl, 2.5 KCl, 1.3 NaH_2_PO_4_, 26 NaHCO_3_, 10 glucose, 2 MgCl_2_, 2 CaCl_2_ (pH 7.3–7.4, osmolarity 300–310 mOsm) for recovery for approximately 30 min at 29–30 °C, and then at room temperature for 1 h. The slices were then transferred to a recording chamber perfused with the ACSF for recording. All solutions were saturated with 95% O_2_/5% CO_2_ prior to use to ensure a stable pH and adequate oxygenation.

Whole-cell recordings were performed at room temperature using glass pipettes (3–5 MΩ) filled intracellular solution containing (mM): 121 KCl, 19 K-Gluconate, 5 NaCl, 4 MgCl_2_, 10 HEPES, 0.1 EGTA, 4 Mg-ATP, Na_2_-GTP (pH 7.3–7.4, osmolarity 280–290 mOsm). Data were collected using a MultiClamp 700B amplifier (Molecular Devices, Sunnyvale CA). Signals were filtered at 2 kHz and digitized at 10 kHz with a Digidata 1550 Data Acquisition System, and analyzed using pCLAMP 10 software (Molecular Devices) and Mini Analysis software (Synaptosoft, Decatur GA).

### Fluorescent immunostaining

Mice were deeply anaesthetized with isoflurane (5% in O_2_) and perfused transcardially with 20 ml PBS followed by 20 ml of cold 4% paraformaldehyde (PFA) in PBS. The whole brain was removed and post-fixed with the same 4% PFA for 4–6 h at 4 °C. The samples were then transferred to 30% sucrose in PBS for at least 48 h in dark. Sample sections (15 mm in thickness) were prepared on gelatin-coated glass slide with a cryostat (Leica). The sections were blocked with 5% goat serum and 0.3% Triton X-100 (Sigma) in TBS buffer for 45 min, and then incubated overnight at 4 °C with primary antibody for rat-anti-CD11b (1:200, Biolegend, Catalogue #101202), rabbit-anti-P2Y12 (1:1000, Anaspec, Catalogue #55043), rabbit-anti-c-Fos (1:500, Cell Signaling, Catalogue #2250). The sections were then incubated for 90 min at room temperature, with secondary antibodies (1:500, Alexa Fluor 488, 594, Life Technologies). The sections were mounted with Fluoromount-G (SouthernBiotech) and fluorescent images were obtained with an EVOS microscope (ThermoFisher). Cell counting and fluorescent signal intensity was quantified using ImageJ software (National Institutes of Health, Bethesda, MD).

### Statistical analysis

Data were presented as Mean ± SEM. Student’s t-test, Wilcoxon rank-sum test (U-test) and two-way ANOVA were used to determine significance.* *p* < 0.05, **p < 0.01, ***p < 0.001.

## Data Availability

The datasets are available from the first author and corresponding author on reasonable request.
